# Practical Points in Children's Feeding

**Published:** 1909-06-05

**Authors:** D. M. MacDonald


### The General Practitioner's Column.

[Contributions to this Column are invited, and if accepted will be paid for.]

### PRACTICAL POINTS IN CHILDREN'S FEEDING.

By D. M. MACDONALD, M.D., D.P.H., Dunkeld.

When it is necessary to hand-feed a baby, it should be remembered that no child under one year, sometimes more, requires undiluted milk. For the first month of life the meal should be in the following proportions: Milk, two tablespoonfuls; barley water, two and a half tablespoonfuls. In the second month three tablespoonfuls of each; in the third, four tablespoonfuls of barley water to five of milk. This applies to healthy children, but if the child is weakly one may give one tablespoonful of milk for each month, diluted.

Besides barley water one may use lime water. The advantage of the former is that, being of a mucilaginous nature, it causes a softer matrix of curd, and it is laxative, whereas the tendency of cow's milk, especially when boiled, is the other way. The proper preparation of barley water is not unimportant. Two teaspoonfuls of prepared barley are mixed with a little water to form a smooth cream, a pinch of salt is added, and then a pint of water. This is poured through a fine strainer into an enamelled saucepan and boiled a quarter of an hour, allowed to cool, then skimmed. (For invalids a little lemon juice added makes a useful demulcent drink.)

Occasionally one has recourse to humanised milk—that is, milk deprived of a proportion of casein with all the fats retained. To prepare it, remove the cream and then divide the milk into two parts, A and B. Treat B with rennet until curdled, bring to the boil, and strain; add B along with the whey to A and the cream. In this way the product contains the sugar and fat in full proportion and the casein in half. Sterilised milk is obtained by heating to 212° F. for one or one and a half hours; pasteurised milk by heating to 155° F. or 170° F. for half an hour. Peptonised milk is obtained by adding the contents of a tube or tablet of pepsin to a certain quantity of milk, which is kept at a warm temperature for 20 minutes; fermentation is then arrested by boiling. If kept longer than 20 minutes before boiling the milk becomes bitter and unpalatable.

It is a good rule to prohibit all food other than milk to children without teeth. The temperature of the food should be 95°, and the feeding-bottle of the boat-shaped order, which is easily kept clean. It is also a good plan to have two bottles and to place the one not in use in a cold solution of boric acid. Sterilised, humanised, peptonised, and pasteurised milks have one point in common: they are all prone to produce anæmia, and should be regarded as temporary expedients.

When the milk constipates, a piece of manna dissolved in the milk once a day or in two days will obviate the necessity for dosing with castor oil, etc. Later on certain adjuncts to food are to be considered. The coarser variety of oatmeal often has the opposite effect desired by worrying the bowels and making them more obstinate, or by causing diarrhœa. Gravy is useful; bread may be steeped in it, or mashed potato. The gravy is best prepared from fresh meat; if from other sources, it should be allowed to stand, the fat skimmed off, then warmed.

Raw-meat juice is very good and may be prepared by scraping the surface of raw meat with a blunt knife, then placing in a jar with some lukewarm water and allowing to stand in a cool place two or three hours. Then take the shredded meat and press through muslin into the water in which it was previously. In hot weather it should be prepared daily.

Milk puddings are excellent, but pastry is to be avoided; mutton broth, too, is useful, and at three years suet puddings. Condensed milk abroad is frequently the only means of obtaining milk. In this country it is more frequently a convenience than a necessity. Given alone for some time it may give rise to rickets; this may, however, be largely or entirely obviated by combining it with mutton broth or beef juice.

